# Dietary Interventions in Pollen-Related Food Allergy

**DOI:** 10.3390/nu10101520

**Published:** 2018-10-16

**Authors:** Sarah A. Lyons, Anne M. van Dijk, André C. Knulst, Eleonora Alquati, Thuy-My Le, Harmieke van Os-Medendorp

**Affiliations:** 1 Department of Dermatology and Allergology, University Medical Center Utrecht, Heidelberglaan 100, 3584CX Utrecht, The Netherlands; A.C.Knulst@umcutrecht.nl (A.C.K.); Eleonora.al@hotmail.it (E.A.); T.T.M.Le-2@umcutrecht.nl (T.-M.L.); H.vanOsMedendorp@umcutrecht.nl (H.v.O.-M.); 2 Laboratory for Translational Immunology, University Medical Center Utrecht, Heidelberglaan 100, 3584CX Utrecht, The Netherlands; 3 Department of Dietetics, University Medical Center Utrecht, Heidelberglaan 100, 3584CX Utrecht, The Netherlands; adijk20@umcutrecht.nl

**Keywords:** pollen-related food allergy, food allergy, pollen, diet, avoidance, immunotherapy, heating, processing, hypoallergenic

## Abstract

In practice, it remains unclear what the best dietary approach is in subjects with pollen-related food allergy (PRFA). Our objective was to evaluate the effect of (1) dietary avoidance advice, (2) oral immunotherapy (OIT), (3) (heat) processing, and (4) consumption of hypoallergenic cultivars on frequency, severity, and eliciting dose of pollen-related food allergic reactions. A systematic search was conducted in PubMed, Embase, and Cochrane. All studies performing an in vivo investigation of one of the four interventions in adults with PRFA were included. Each study was assessed for quality and validity. Available data on frequency, severity, and eliciting dose of allergic reactions were extracted. Ten studies matched the eligibility criteria. No studies were retrieved on dietary avoidance advice. Two studies (*N* = 92) on apple OIT reported that tolerance was induced in 63% and 81% of subjects. Four studies (total *N* = 116) focused on heat processing. Heating was found to completely eradicate symptoms in 15–71% of hazelnut allergic and 46% of celery allergic individuals. Four studies (*N* = 60) comparing low to high allergenic apple cultivars revealed that Santana (and possibly Elise) apples seemed to cause milder reactions than Golden Delicious. In the awareness that overall level of evidence was low, we conclude that OIT, heat processing, and hypoallergenic cultivars may diminish or completely prevent allergic reactions in some but not all subjects with PRFA.

## 1. Introduction

Up to 90% of pollen-sensitised individuals are allergic to foods that cross-react with pollen [1,2,3,4]. This pollen-related food allergy (PRFA) is generally characterised by the rapid onset of oropharyngeal symptoms after ingestion and spontaneous resolution within 30 min; systemic reactions are possible but rare [5,6]. Birch PRFA is most common in Northern and Central Europe, but food allergy due to cross-reactivity with mugwort, grass, and plant weed is also described [5,6]. Frequently involved foods include *Rosaceae* fruits (e.g., apple, peach, cherry), *Apiaceae* vegetables (e.g., carrot, celery), peanut, tree nuts, and soybean [5,6]. The increasing incidence of pollen allergy will probably lead to a further increase in PRFA [5,6,7,8,9].

Primary dietary therapy for food allergy consists of the avoidance of triggering foods [5,6,10,11]. However, clinical guidelines on PRFA give no specific advice regarding avoidance of cross-reacting foods or foods within the same family, nor with regard to avoidance of traces [5,6]. As a result, the specifics of the avoidance recommendations differ per physician. In a survey of Ma et al., 9% of US allergists did not impose any diet restrictions, 53% of allergists advised avoidance of triggering foods, 4% recommended avoiding potential cross-reacting foods, and 38% based their treatment on individual patient presentation [12]. It remains unclear what the effect of these varying treatments is on pollen-related food allergic reactions in practice.

Furthermore, the clinical efficacy of other dietary interventions, such as oral immunotherapy (OIT) with food, heat processing, and consumption of low allergenic cultivars on PRFA is unknown.

Whereas current guidelines do not recommend pollen immunotherapy to treat PRFA, no guidance is given regarding OIT with food [5,6,10]. A recent study investigating the effectiveness of sublingual immunotherapy with recombinant Mal d 1 allergen extract in pollen-related apple allergic patients, showed that this type of immunotherapy was a safe and effective approach to reduce symptoms [13]. A more practicable dietary therapy comprising OIT with the culprit food, has been systematically evaluated and found to be effective in treating allergy to milk, egg, wheat and peanut [14,15]. Although peanut allergy can be a PRFA due to cross-reaction between birch pollen and peanut component Ara h 8 primarily [5,16], the efficacy of OIT was not specifically discussed for such subjects, and the role of oral immunotherapy with food in PRFA is still unclear.

Clinical guidelines describe that heat processing the culprit food can reduce PRFA symptoms, because major food allergens cross-reacting with tree pollen are heat labile [5,6]. Skin prick tests (SPT) in subjects with PRFA are less often positive with cooked than with raw culprit foods [17]. The extent of skin test positivity also appears to depend on the amount of allergen content in different cultivars of the culprit food [5,6] which gives the impression that consumption of low allergenic rather than high allergenic cultivars may be a valuable dietary intervention. However, the effect of heating or consumption of low allergenic cultivars on the allergic symptoms of subjects with PRFA remains to be evaluated.

Therefore, the aim of this review was to evaluate the effect of specific dietary interventions on frequency, severity, and eliciting dose of food allergic reactions in adults with PRFA. Evaluated dietary interventions consisted of (1) dietary avoidance advice, (2) OIT with food, (3) (heat) processing, and (4) consumption of hypoallergenic cultivars. 

## 2. Materials and Methods

### 2.1. Protocol and Registration

This systematic literature review was carried out according to a protocol registered in advance in the international prospective register of systematic reviews (PROSPERO), registration number CRD42018103805, and presented following the recommendations of the PRISMA checklist [18].

### 2.2. Eligibility Criteria, Information Sources, and Search

Relevant synonyms for our domain (adults with PRFA) and determinants (dietary avoidance advice, OIT with food, heat processing, and consumption of hypoallergenic cultivars) were combined to develop an extensive search strategy (Appendix A), which was entered into PubMed, Embase, and The Cochrane Library on 6 July 2018 using keywords and Medical Subject Headings. 

A broad search terminology for PRFA was used as well as particular terms for relevant plant-related inhalant allergens and for specific foods reported to cross-react with these inhalant allergens in recent position papers by European allergy working groups [5,6].

With regard to dietary avoidance advice, we aimed to find studies on the efficacy of different types of dietary advice in practice. Three predetermined dietary interventions were additionally incorporated in the search: OIT, (heat) processing, and consumption of hypoallergenic cultivars. No study design, date or language restrictions were imposed.

### 2.3. Study Selection

After importation of all identified citations into EndNote and removal of duplicates, title and abstract screening, and subsequent full text screening were performed by two independent authors (E.A., A.M.v.D.). Selection was based on consensus; any discrepancies were resolved by consultation of other reviewers (S.A.L., H.v.O.-M., and T.-M.L.). In case of full text unavailability, we attempted to contact authors via email. References of selected articles, reviews and meta-analyses were hand searched and checked in the Scopus citation database for additional articles of interest.

All articles in English, Dutch, German, French, Spanish, and Italian were assessed. For inclusion, the study population had to meet three criteria: (1) ≥80% of the participants were 18 years or older; (2) subjects had a convincing history of hay fever or a positive SPT or ImmunoCAP to at least one type of pollen extract; and (3) subjects had a history of allergic reactions to foods known to cross-react with pollen as well as sensitisation (SPT or CAP) or positive challenge test to the food concerned. Studies were further assessed if they investigated at least one of the determinants of interest. 

Studies evaluating immunotherapy other than OIT with eliciting food were excluded, as were studies where low allergenic cultivars were not compared to high allergenic cultivars. We also eliminated non-original studies (reviews, editorials, and expert opinions), conference abstracts, case studies, animal studies, post-mortem studies, etiologic, diagnostic, and prognostic studies, in vitro studies, and in vivo studies where allergy was only evaluated by SPT. 

### 2.4. Data Collection

Two authors (A.M.v.D., S.A.L.) independently collected and recorded study characteristics on a predefined checklist, comprising the items author, setting, time frame, study design, study population, method of intervention, method of outcome measurement, and reported outcomes. In some studies, we evaluated part of the total study population because outcomes regarding our determinants of interest were only available for a subgroup of subjects.

For OIT, we obtained data regarding the frequency of achieved tolerance and tolerated dose at final follow-up. For processing and consumption of hypoallergenic cultivars, data on the number of subjects with no allergic reactions after intervention, on symptom severity, and on the eliciting dose were extracted. In order to improve comparability of results from individual studies, the proportion of subjects with an allergic reaction, the median VAS score for symptom severity, and the median dose eliciting symptoms were calculated from available data where possible. 

### 2.5. Risk of Bias Assessment

The validity of included studies was assessed for the part of the study population considered relevant for our research question. The Robins-I tool [19] was used to evaluate seven potential sources of bias: bias due to confounding, bias in selection of participants into the study, bias in classification of interventions, bias due to deviations from intended interventions, bias due to missing data, bias in measurement of outcomes, and bias in selection of the reported results. Two authors (A.M.v.D., S.A.L.) performed an independent evaluation and discussed disagreements to reach consensus. Each article received a final risk level of “low risk”, “moderate risk”, “high risk”, “critical risk”, or “no information”.

### 2.6. Synthesis of Results

Because of evident heterogeneity in methodology and reporting between studies, it was considered inappropriate and infeasible to pool results. A qualitative synthesis of available results was therefore performed. No statistical analyses were conducted.

The overall level of the evidence per study outcome per intervention of interest was assessed using the GRADE system [20] and categorised as high quality, moderate quality, low quality or very low quality.

## 3. Results

### 3.1. Study Selection

**Figure 1:**
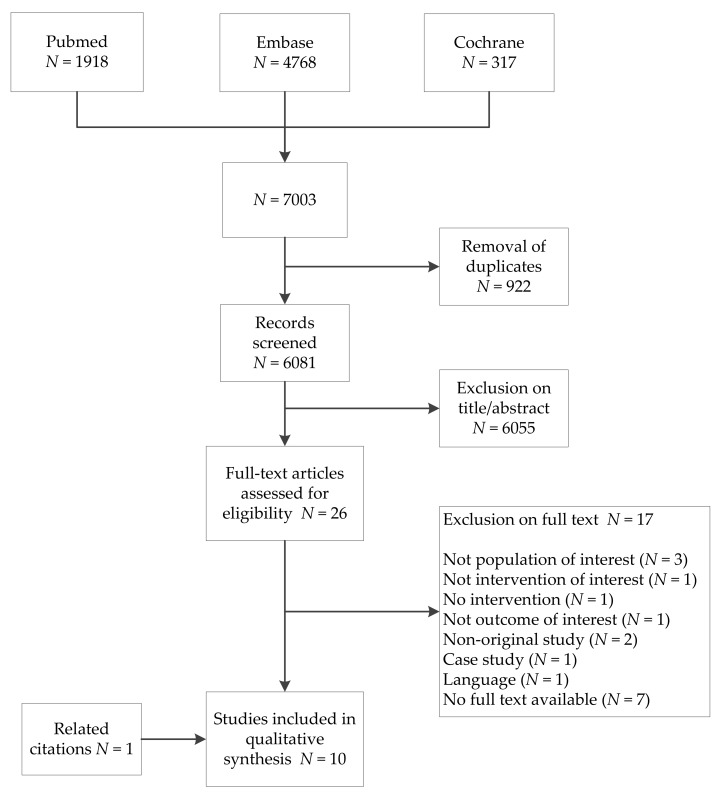
Flowchart.

Our search yielded 6081 unique citations (Figure 1). Screening of title, abstract, full-text, and related citations provided ten articles suited to address our research question, including one article found via reference checking. 

### 3.2. Study Characteristics

Details of the ten selected studies can be found in Table 1. They were all conducted in Western Europe and published in French [21] or English [22,23,24,25,26,27,28,29,30].

All included subjects reported allergy to apple, hazelnut, celery, or carrot and had a history of pollen allergy or were sensitised to birch pollen (and additionally mugwort pollen in one study [23]).

No studies were obtained regarding the effect of dietary avoidance advice on frequency, severity, and eliciting dose of allergic reactions. 

Two studies, including one randomized controlled trial (RCT), focused on OIT with increasing doses of Golden Delicious apple [21,22] in a total of 92 subjects. Both reported the number of subjects in the intervention group that achieved tolerance to apple and that could consume other *Rosaceae* fruits after a follow-up period of respectively 48 weeks [21] and 8 months [22]. One study provided information on median tolerated dose [22]. Neither study evaluated permanent tolerance, generally referred to as sustained unresponsiveness after a period of discontinuation of regular apple consumption [14]. No studies were found to evaluate OIT with other foods in our study population of interest. 

In four studies with 116 subjects in total, authors reported on the effect of heat processing of hazelnut [25,26], celery [23,24], carrot [24] and apple [24]. In order to measure the effect, reactions in double-blind placebo-controlled food challenge (DBPCFC) with heated food was compared to reactions to raw food in DBPCFC or history. One study also investigated the effect of processing to celery spice on allergenicity of celery in this patient population [23]. The number of subjects with an allergic reaction to the processed food and their specific symptoms were reported in all studies, along with the information on the tolerated dose in three studies [23,25,26]. Other than heat processing and processing to celery spice, no other methods of processing appeared to have been evaluated in vivo by comparative raw versus processed food challenge. 

Four studies compared the allergenicity of putatively high allergenic to putatively hypoallergenic apple cultivars, primarily assessing the difference in severity of allergic reactions by single- or double-blind food challenge in 60 subjects altogether [27,28,29,30]. Golden Delicious (GD), which was classified as the high allergenic cultivar in all studies, was compared to Santana apple in three studies [28,29,30], and Elise [30], Pink Lady [30], Topaz [29] and G-198/Orim [27] apples in one study each. All studies used various visual analogue scales to assess severity of reactions. Three studies provided information on the proportion of subjects who remained free of symptoms to the various apple cultivars [27,29,30]. The dose eliciting symptoms was discussed in only one study [28]. No studies were found to compare low to high allergenic cultivars for other foods than apple in subjects with PRFA.

### 3.3. Risk of Bias Assessment

The risk of bias in relation to our study question was moderate to high for all included studies, mainly due to possible confounding, selection bias (generally because only a subgroup of the total study population in some studies was relevant for this review), and bias in outcome measurement. Details of the assessment are presented in Table 2.

### 3.4. Synthesis of Results and Level of Evidence

A summary of our findings is found in Table 3, Table 4 and Table 5.

#### 3.4.1. Oral Immunotherapy

After OIT with Golden Delicious apple, tolerance to apple was achieved in 63–81% of subjects (Table 3). In an RCT, Kopac et al. found the frequency of achieved tolerance after 8 months to be significantly higher in the intervention than in the control group (63% vs. 0%, *p* = 0.0001). In this study, authors also showed that the median tolerated dose was significantly higher at final follow-up compared to start of study in responders to OIT (*N* = 17, difference in median tolerated dose = 126 g, *p* = 0.0009), in contrast to the controls (*N* = 13, difference in median tolerated dose = 0 g) [22].

Tolerance to other cross-reactive fruits, vegetables, and nuts was reported to varying degrees (14–29%) in the OIT group by Kopac et al. [22]. Bouvier et al. stated that 98% of subjects who achieved tolerance to apple were able to eat other *Rosaceae* fruits (Table 3) [21]. 

Overall, OIT with apple appears effective in inducing tolerance to apple and some cross-reacting foods in individuals with PRFA. Level of evidence for these findings was very low according to GRADE-assessment (Table 6).

#### 3.4.2. (Heat) Processing

In subjects with challenge-confirmed allergy to raw hazelnut, the percentage of subjects who were completely tolerant to roasted hazelnut varied from 15 to 71% amongst the two included studies (Table 4). Symptoms to both raw or roasted hazelnut were only mild, but the median dose required to elicit symptoms with roasted hazelnut appeared higher than with raw hazelnut [25,26]. 

For celery, Ballmer et al. found that 46% of subjects experienced no symptoms to cooked celery and no subjects (0%) tolerated celery spice. Six of 12 (50%) subjects had a moderate to severe reactions to raw celery, one of 11 (9.1%) to cooked celery and three of five (60%) to celery spice [23]. Only one case of celery allergy was examined by Bohle et al. and this subject had mild symptoms to raw and no symptoms to cooked celery [24]. There was insufficient information to compare dose thresholds between raw and processed celery.

Carrot was evaluated in three subjects and apple in one subject [24]. All subjects had mild symptoms to raw carrot or apple and no symptoms to cooked carrot or apple. No conclusions could be drawn regarding eliciting dose. 

Overall, four studies on heat processing (mainly of celery and hazelnut) found that 15–100% of subjects with challenge-confirmed allergy to raw food experienced no symptoms to the same food when heated. GRADE-assessment resulted in a very low level of evidence for each of the evaluated outcomes (Table 6).

#### 3.4.3. Hypoallergenic Cultivars

As described in Table 5, the percentage of subjects who remained completely asymptomatic after the final dose was described to be significantly higher for Santana apple than for Golden Delicious or Topaz apple by Kootstra et al. (54% vs. 7% vs. 7% respectively, *p* = 0.002) [29], but did not differ significantly between Santana, Golden Delicious, Elise, and Pink Lady apple according to Vlieg et al. [30], nor between Golden Delicious and G-198/Orim in Asero et al. [27].

All studies evaluating allergenicity of Santana apple showed that the symptom severity score after challenge with Santana apple was significantly lower than after challenge with Golden Delicious apple in subjects with pollen-related apple allergy (*p* < 0.05) [28,29,30]. Santana apple was also reported to be significantly less allergenic than Topaz apple in one study (*p* = 0.004) [29]. Vlieg et al., who compared severity of symptoms caused by Golden Delicious apple to those caused by Santana, Elise, and Pink Lady apples, conclude that Elise is also a low allergenic apple cultivar for subjects with PRFA [30]. On comparison of G-198/Orim to Golden Delicious apple in this patient population, both cultivars were found to cause the most severe reaction equally often [27]. 

Regarding the dose eliciting symptoms, Bolhaar et al. found that the quantities needed to provoke a reaction of equal severity were on average 30 times higher for Santana than for Golden Delicious apples (*p* < 0.001) [28]. Other studies did not report on this outcome [27,29,30]. 

Altogether, studies comparing low to high allergenic apple cultivars showed that Santana (and possibly Elise) apples seemed to cause milder allergic reactions than Golden Delicious apples in PRFA. The quality of evidence for the three investigated outcomes was graded as very low for the effect of this intervention.

## 4. Discussion

Overall, robust evidence regarding the effect of dietary interventions on the frequency, severity and eliciting dose of allergic reactions in subjects with PRFA is lacking. Evidence regarding the effect of specific dietary avoidance advice in this population is completely absent. Nonetheless, taking the low level of evidence into account, this systematic review of the available literature suggests that certain dietary treatments or adjustments can be beneficial for this group of patients. First of all, OIT with Golden Delicious apple seems to be effective in reducing the frequency of allergic reactions in subjects with birch pollen-related apple allergy, inducing tolerance in 63–81% of subjects. Secondly, heating of foods cross-reacting with birch or mugwort pollen appears to reduce allergenicity in subjects with PRFA, leading to complete prevention of allergic symptoms in 15–100%. Heating also possibly increases the dose threshold for symptom elicitation in pollen-related hazelnut allergic subjects. Finally, Santana and possibly Elise apples seem to cause less severe allergic reactions than Golden Delicious apples in subjects with birch pollen-related apple allergy.

### 4.1. Oral Immunotherapy

OIT with apple was found to result in tolerance in 63% [22] and 81% [21] of subjects, a varying but high response rate. Previous studies on the effect of OIT with plant-based foods were mainly focused on peanut [14,15] and were not included in this review because they were performed mainly in children and without the inclusion criterion of pollen allergy. However, these studies on peanut OIT showed that the rate of tolerance was found to range similarly to our review from 61–100% [14,15]. Sustained unresponsiveness in peanut studies, characterised by absence of symptoms to peanut despite irregular intake or prolonged avoidance, was achieved less frequently in 30–78% [14,15]. Although no evaluation of sustained unresponsiveness was performed for OIT with apple in the studies included in this review, Kopac et al. also suggest that tolerance may be transient, because no significant immunologic changes were observed and one subject experienced a relapse after discontinuing apple consumption during a holiday [22]. Therefore, OIT with apple in subjects with pollen-related apple allergy may be effective, but regular consumption after completion of the study is likely necessary to maintain tolerance. 

Both Kopac et al. and Bouvier et al. reported that the majority of subjects with pollen-related apple allergy could consume other fruits and nuts after OIT with apple [21,22]. An explanation could be that these cross-reactive birch pollen-related foods share homologous aminoacid sequences, and therefore allergenic epitopes on the surface of these homologues [31]. Desensitisation to these epitopes in apple might result in desensitisation to these epitopes in other cross-reacting foods, inducing tolerance to more foods than just apple.

### 4.2. (Heat) Processing

The effect of heating on clinical presentation of PRFA has mainly been investigated for hazelnut and celery, and was found to eradicate symptoms in 15–71% of hazelnut and around 46% of celery allergic subjects. Furthermore, roasting of hazelnut resulted in higher dose thresholds [25,26] and boiling of celery caused fewer moderate to severe reactions [23]. Sensitisation to the birch pollen- related PR-10 proteins, which are heat labile, explains the symptom diminishing effect of heat processing [32]. However, the effect of heating does not appear to be equivalent for all Bet v 1 homologues [24,32]. Where apple Mal d 1 undergoes a continuous unfolding process during thermal processing, carrot Dau c 1 and celery Api g1 do not begin to change structure until higher temperatures (respectively 28 °C, 43 °C, and 50 °C) [24]. Furthermore, Api g 1 returns to its native structure after recooling, where Mal d 1 and Dau c 1 do not [24]. Hazelnut Cor a 1 is reported to be heat-resistant below 100 °C [33]. These findings imply that, although heating may reduce symptoms in subjects with birch PRFA, this effect differs depending on the food. 

However, not only the level of heating appears to influence the allergenicity of pollen-related foods, as heating at the same temperature depleted allergenicity in some subjects but not in others. An explanation is that subjects may also be sensitised to heat-stable allergens, such as lipid transfer proteins (e.g., hazelnut Cor a 8, celery Api g 2) or seed storage proteins (e.g., hazelnut Cor a 9 and 14) [23,33,34]. (Co-) sensitisation to these allergens may explain why some subjects reacted to cooked celery [23] or roasted hazelnut [25,26]. In fact, Ballmer et al. provide support for this statement by demonstrating that not all celery allergic subjects were sensitised to the birch pollen-related Api g 1. Another explanation could be that the effect of heat processing foods in pollen-allergic subjects depends on the type of pollen sensitisation. For example, one study demonstrated that celery-mugwort sensitised subjects were IgE-sensitised to heated celery, whereas celery-birch sensitised subjects were not [35], indicating that mugwort-sensitised subjects may be more likely to react to heated celery. 

Processing to spice does not appear to have the same effect as heat processing, but this was only studied for celery. All celery allergic subjects who underwent challenge with celery spice were found to be allergic to celery spice as well as to raw celery [23]. A previous in vitro study by Jankiewizc et al. also showed that it is possible to detect Api g 1, Api g 4 and celery CCD by specific IgE antibodies in celery spice [17], which supports the in vivo findings by Ballmer-Weber et al. Therefore, celery spice is not safe for pollen-related celery allergic subjects.

### 4.3. Consumption of Hypoallergenic Cultivars

To date, research with regard to the effect of consumption of alternative hypoallergenic cultivars on clinical symptoms in subjects with PRFA has focused on apple, showing that Santana apple appears to cause significantly less severe reactions than Golden Delicious apple [28,29,30]. These findings were later strengthened in a non-clinical setting, where around 40% of consumers with mild to moderate self-classified apple allergy reported having no symptoms to Santana apple [36]. Another apple which could be considered clinically preferable in this patient population based on the results of this review are Elise apples [30]. 

However, there were also some differences between similar apple cultivar comparisons in the different studies. For example, in Kootstra et al. subjects reached DBPCFC final dose (±100 g) of Santana apple significantly more often than of Golden Delicious apple [29], whereas no significant difference was found between the same cultivars in Vlieg et al. (final dose 120 g) [30]. Other factors to take into consideration which may influence severity of allergic reactions to apple are season [37], storage [28,30,38], consumption with or without peel [39], and intra-cultivar variation [27]. These elements were not part of this review, however.

It also becomes clear that classification of apple as hypoallergenic based on SPT [28,30] or Mal d 1 content [27] does not imply equally reduced symptomatology compared to high allergenic apples like Golden Delicious. Although Santana, Topaz, Pink Lady, and Elise were all classified as low allergenic [28,30], VAS scores of Santana were significantly lower than those of Topaz and VAS scores of Elise were significantly lower than those of Santana and Pink Lady [29,30]. Neither SPT nor Mal d 1 content seem to predict allergenicity of different apple cultivars as determined by food challenge [40].

### 4.4. Strengths and Limitations

In evaluating this review, the reader should remain aware of the low level of evidence due to small sample sizes, suboptimal study designs, and heterogeneity in intervention and outcome reporting between studies. The latter aspect discouraged pooling and meta-analysis. We would also like to point out that several studies had different primary aims than our review question. This meant that we had to focus on subgroups that dealt with our research question in some studies, possibly introducing selection bias as not all characteristics of the selected subgroups were available [23,26,27]. Finally, we were unable to find any studies in which the effect of dietary avoidance advice in practice on the frequency and severity of allergic reactions was evaluated. 

Nonetheless, this is the first review analysing PRFA from a dietary point of view, in which we present an overview of potentially relevant dietary interventions to aid physicians, dietitians, and nutritionists in advising and treating these patients in practice. We feel our broad research question, extensive search strategy, transparent critical appraisal, and concise presentation of study characteristics and results will allow readers to make a conscious appreciation and interpretation of the available information. 

## 5. Conclusions

In conclusion, subjects with pollen-related apple allergy may benefit from OIT with apple, which may additionally reduce symptoms to cross-reactive foods. Furthermore, apple allergic patients can expect less severe reactions if they consume the hypoallergenic apple cultivars Santana or Elise. Additionally, thermal processing of causative foods in subjects with PRFA likely reduces symptoms, but the effect size may depend on the food concerned. These findings can be used to advise subjects with PRFA on their diet, taking into account that the level of evidence is low. 

In the knowledge that up to 90% of pollen-sensitised individuals suffer from PRFA [1,2,3,4], which can cause symptoms to a wide variety of fruits, nuts, and vegetables and thus deprive these individuals of valuable sources of vitamins, minerals, and fibre, more dietary intervention studies are necessary to consolidate our findings and evaluate the effect of avoidance versus allowance of causative foods, traces of causative foods and cross-reactive foods in the diet of patients with PRFA.

## Figures and Tables

**Table 1:** Study characteristics.

Study Information	Study Design	Relevant Study Population	Method of Intervention	Method of Outcome Measurement	Outcomes Reported
Oral Immunotherapy					
Bouvier et al.; Lyon hospital allergy clinic (F); 1 May 2012–1 February 2013 [21]	NS, comparison of participants’ allergic reactions before and after oral immunotherapy	52 subjects (age 8–63 years; 17% <18 years) with IgE-sensitisation to birch pollen and apple; and OAS to *Rosaceae* foods according to history	All subjects underwent oral immunotherapy with increasing doses of fresh Golden Delicious (GD) apple. 1. Initial dose escalation with nine doses from 0.1 mg to 16 g. Portion increased every 20–30 min. 2. Build-up phase starting at 16 g and increasing to half an apple (64 g) 3 times per week up until 24 weeks after start. 3. Maintenance phase consisting of half an apple 3 times a week up until 48 weeks after start.	Patient history at 48 weeks follow-up	1. Number of subjects that achieved tolerance of 64 g of apple after 48 weeks. 2. Number of subjects that was tolerant to other *Rosaceae* fruits after oral immunotherapy with apple.
Kopac et al.; University hospital Bern allergy clinic (CH); December 2009–August 2010 [22]	RCT	40 subjects (age 18–61 years) with IgE-sensitisation to birch pollen and Mal d 1; and challenge-confirmed OAS to Golden Delicious apple	27 of 40 subjects underwent oral immunotherapy with increasing doses of fresh Golden Delicious (GD) apple. 1. Initial dose escalation with doses from 1 g to 128 g. Portion doubled every 5 min. 2. Build-up phase starting at the largest dose tolerated in the preceding phase to whole apple (150–200 g). Portion doubled every 2–3 weeks. 3. Maintenance phase commencing when a whole apple was tolerated (average 20 weeks) and consisting of at least three apples per week up until 8 months after the start. 13 of 40 subjects remained untreated and formed the control group.	Patient history at 8 months follow-up	1. Proportion of subjects that achieved tolerance to 128 g of apple after 8 months. 2. Number of subjects that achieved cross-tolerance to other birch pollen cross-reacting fruits/nuts after oral immunotherapy with apple.
**(Heat) Processing**					
Ballmer-Weber et al.; University hospital Zurich allergy clinic (CH); January 2000–February 2001 [23]	NS, comparison of participants’ allergic reactions to processed and unprocessed variants of the food	12 subjects (age 21–42 years) with IgE- sensitisation to birch pollen (and mugwort pollen in 9/12 subjects) and celery; and allergic reactions to celery according to history	1. Cook celery (110 °C; 15 min) 2. Dehydrate celery (celery spice)	Comparison of DBPCFC with processed celery (12 of 12 subjects) to DBPCFC with raw celery (10 of 12 subjects) or convincing history to raw celery (2 of 12 subjects)	1. Number of subjects with symptoms in response to oral challenge with cooked celery and celery spice 2. Type of symptoms 3. Dose eliciting symptoms
Bohle et al.; Hannover Medical School Department of Dermatology and Allergology (D); *time NS* [24]	NS, comparison of participants’ allergic reactions to processed and unprocessed variants of the food	5 subjects (age 5–37 years; 20% <18 years) IgE-sensitised to birch pollen with OAS and worsening of atopic dermatitis to carrot, celery or apple according to history	Celery (1 of 5 subjects): boil until soft Carrot (3 of 5 subjects): boil until soft Apple (1 of 5 subjects): pasteurisation (juice)	Comparison of DBPCFC with processed food (4 of 5 subjects) or convincing history to processed food (1of 5 subjects) to DBPCFC with raw food (5 of 5 subjects)	1. Number of subjects with OAS in response to oral challenge with cooked carrot or celery or apple 2. Type of symptoms
Hansen et al.; University hospital Copenhagen (DK), and University hospital Zurich allergy clinics (CH); 1998–2000 [25]	NS, comparison of participants’ allergic reactions to processed and unprocessed variants of the food	17 subjects (age 14–65 years) with IgE-sensitisation to birch pollen and hazelnut; and OAS to hazelnut according to history or challenge	Roast hazelnut (140 °C; 40 min)	Comparison of DBPCFC with roasted hazelnut (17 of 17 subjects) to DBPCFC with raw hazelnut (16 of 17 subjects) or convincing history to raw hazelnut (1 of 17 subjects)	1. Number of subjects with symptoms in response to oral challenge with roasted hazelnut 2. Type of symptoms 3. Dose eliciting symptoms
Worm et al.; University hospital Charité Berlin dermatology outpatient clinic (D); *time NS* [26]	NS, comparison of participants’ allergic reactions to processed and unprocessed variants of the food	82 of 132 included subjects (age 21–65 years) with IgE-sensitisation to birch pollen and hazelnut; and challenge-confirmed hazelnut allergy	Roast hazelnut (144 °C; time unknown)	Comparison of DBPCFC with roasted hazelnut (20 of 82 subjects) to DBPCFC with raw hazelnut (82 of 82 subjects)	1. Number of subjects with symptoms in response to oral challenge with roasted hazelnut 2. Type of symptoms 3. Dose eliciting symptoms
**Consumption of Hypoallergenic Cultivars**					
Asero et al.; *setting NS*; 2004 [27]	NS, comparison of participants’ allergic reactions to low and high allergenic cultivars	7 of 17 included subjects (age 26–49) with sensitisation to birch pollen and apple; and OAS to apple according to history	Consumption of low allergenic G-198 or Orim apple	Comparison of SBFC with G-198 apple (6 of 7 subjects) or Orim apple (1 of 7 subjects) to SBFC with Golden Delicious apple	1. Mean symptoms severity score for OAS (Score 0–100) 2. Number of subjects reporting NO symptoms in response to oral challenge
Bolhaar et al.; University Medical Centre Utrecht department of dermatology and allergology (NL); *time NS* [28]	NS, comparison of participants’ allergic reactions to low and high allergenic cultivars	5 of 23 included subjects (age > 18 years) with a history of rhinoconjunctivitis during birch pollen season, sensitisation to apple, and OAS to apple according to history	Consumption of low allergenic Santana apple	Comparison of DBPCFC with Santana apple to DBPCFC with Golden Delicious apple	1. Mean symptom severity score for OAS (VAS 0–100) 2. Quantities needed to provoke similar VAS score for Santana apples as for Golden Delicious apples
Kootstra et al.; University Medical Centre Groningen allergy outpatient clinic (NL); February–May 2005 [29]	NS, comparison of participants’ allergic reactions to low and high allergenic cultivars	15 subjects (age > 18 years) with sensitisation to birch pollen and apple; and challenge-confirmed OAS to apple	Consumption of low allergenic Santana apple	Comparison of SBFC with Santana apple to SBFC with Golden Delicious apple as a positive control and SBFC with Topaz apple as a negative control	1. Maximum symptom severity score (VAS, range not described) at dose 1. 2. Number of subjects reporting NO symptoms in response to oral challenge.
Vlieg-Boerstra et al.; University Medical Centre Groningen allergy outpatient clinic (NL); 2006–2008 [30]	NS, comparison of participants’ allergic reactions to low and high allergenic cultivars	33 subjects (age 18–52 years) with sensitisation to birch pollen in 32/33 subjects; and challenge-confirmed OAS to apple	Consumption of low allergenic Elise, Santana and Pink Lady apples	Comparison of SBFC with Elise, Santana, Pink Lady and Golden Delicious apple	1. Cumulative symptom severity score (VAS, range not described) at dose 1. 2. Number of subjects reporting NO symptoms in response to oral challenge.

F = France, CH = Switzerland, D = Germany, DK = Denmark, NL = The Netherlands; NS = not specified; RCT = Randomised controlled trial; OAS = oral allergy syndrome; DBPCFC = double-blind placebo-controlled food challenge; SBFC = single-blind food challenge; VAS = Visual analogue scale.

**Table 2:** Risk of bias assessment *.

	Confounding	Selection	Classification of Interventions	Deviations from Interventions	Missing Data	Outcome Measurement	Selection of Reported Results	Overall Risk
Bouvier et al. [21]	●	●	●	●	●	●	●	●
Kopac et al. [22]	●	●	●	●	●	●	●	●
Ballmer-Weber et al. [23]	●	●	●	●	●	●	?	●
Bohle et al. [24]	●	●	●	●	●	●	●	●
Hansen et al. [25]	●	?	●	●	●	●	●	●
Worm et al. [26]	●	●	●	●	●	●	●	●
Asero et al. [27]	●	●	●	●	●	●	●	●
Bolhaar et al. [28]	●	●	●	●	●	?	●	●
Kootstra et al. [29]	●	●	●	●	●	●	●	●
Vlieg-Boerstra et al. [30]	●	●	●	●	●	●	●	●

* The Robins-I tool was used for Risk of Bias assessment [19]; ● = low risk of bias; ● = moderate risk of bias; ● = high risk of bias; ? = unclear risk of bias.

**Table 3:** Summary of findings–oral immunotherapy with Golden Delicious apple.

Source	Number of Subjects	Build-Up Phase Completed *N* (%)	Maintenance Phase Completed *N* (%)	Frequency of Achieved Tolerance at Final Follow-Up *N* (%) *	Tolerated Dose	Frequency of Achieved Tolerance to Other Birch Pollen Cross-Reacting Foods
Bouvier et al. [21]	52 Active: 52 Control: NA	46/52 (88.5) Week 24; 1 non-responder; 5 drop-outs	42/52 (80.8) Week 48; 4 missings	42/52 (80.8)	No information	41/42 subjects reported tolerance to other *Rosaceae* fruits (cherries, peaches), kiwi fruit, nuts, and peanuts. One apple-tolerant subject reported being unable to consume carrots.
Kopac et al. [22]	40 Active: 27 Control: 13	17/27 (63.0) Week 20 (range 7–30); 5 non-responders; 5 drop-outs	17/27 (63.0) Month 8 (T8); 0 missings	Active: 17/27 (63.0) Control: 0/13 (0.0) *p* (active vs. control) = 0.0001	Active: Responders (*N* = 17): Median tolerated dose Δ_T8-T0_: 126 (69–127) g *p* (Δ_median tolerated dose_) = 0.0009 Non-responders (*N* = 5): Median tolerated dose Δ_T8-T0_: 8 (0–60) g *p* (Δ_median tolerated dose_) = NA Control: Median tolerated dose Δ_T8-T0_: 0 (−24–6) g*p* (Δ_median tolerated dose_) = NA	Of subjects who reported symptoms to cross-reactive fruits in the active group and who completed protocol, 29% could tolerate pear where they could previously not. 27% could tolerate cherries, 23% hazelnuts, 14% walnuts, 18% peaches. In the control group 1 patient could tolerate pear where he could previously not; no other changes were observed.

NA = not available; Responders = Subjects who successfully reached maintenance dose following OIT protocol; Non-responders = Subjects who could not successfully reach maintenance dose despite following OIT protocol; * Intention-to-treat analyses.

**Table 4:** Summary of findings—(heat) processing.

Source	Food and Number of Subjects	Frequency of NO Symptoms in DBPCFC with Processed Food *N* (%)	Frequency of Symptoms in DBPCFC with Processed Food *N* (%)	Symptom Severity in DBPCFC with Raw vs. Processed Food	Eliciting Dose in DBPCFC with Raw vs. Processed Food
Ballmer-Weber et al. [23]	Cooked celery: 11 Celery spice: 5	Cooked celery: 5/11 (45.5) Celery spice: 0/5 (0)	Cooked celery: 6/11 (54.5) Celery spice: 5/5 (100.0)	Raw celery: 6× mild, 3× moderate, 3× severe Cooked celery: 5× no symptoms, 5× mild; 1× severe Celery spice: 2× mild; 3× moderate	Raw celery: 7× 0.7 g; 3× 28.5 g Cooked celery: 3× 0.9 g, 2× 1.8 g, 1× 34.5 g Celery spice: 3× 0.16 g, 1× 0.32 g, 1× 5.85 g *
Bohle et al. [24]	Carrot: 3 Celery: 1 Apple: 1	Cooked food: 5/5 (100.0)	Cooked food: 0/5 (0.0)	Raw food: 5× mild Cooked food: 5× no symptoms	No information
Hansen et al. [25]	Hazelnut: 17	Roasted hazelnut: 12/17 (70.6)	Roasted hazelnut: 5/17 (29.4)	Raw hazelnut: 17× mild Roasted hazelnut: 12× no symptoms, 5× mild	Raw hazelnut: Median dose Copenhagen (*N* = 10) 2 g; Zurich (*N* = 7) 2.6 g Roasted hazelnut: Median dose Copenhagen (*N* = 4) 7 g; Zurich (*N* = 1) 5.2 g *p* (roasted vs. raw) = NA
Worm et al. [26]	Hazelnut: 82	Roasted hazelnut: 3/20 (15.0)	Roasted hazelnut: 17/20 (85.0)	Raw hazelnut: 78× mild, 4× unclear Roasted hazelnut: 17× mild	Raw hazelnut: Median dose 0.1 g, range 0.01–2.0 g Roasted hazelnut: Median dose 0.23 g, range 0.01–10.0 g *p* (roasted vs. raw) = 0.009

NA = Not available; Mild = oral allergy symptoms, rhinitis, conjunctivitis, pruritis; Moderate = urticaria, angioedema, flush, vertigo, gastro-intestinal symptoms; Severe = dyspnea, collapse; * Protein content of celery spice is 4.5 times as high as protein content raw celery.

**Table 5:** Summary of findings—alternative hypoallergenic cultivars.

Source	Number of Subjects	Food	Frequency of NO Symptoms to Highest Dose in FC *N* (%)	Symptom Severity in FC with Various Cultivars	Dose Eliciting Symptoms in FC with Various Cultivars
Asero et al. [27]	7	High allergenic Golden Delicious vs. low allergenic G-198/ Orim	GD: 0/7 (0.0) G-198/Orim: 0/7 (0.0) (*p* = NA)	GD: median VAS score 2/10; range 2–8. G-198/Orim: median VAS score 4/10; range 2–8. 2/7 patients reported more severe OAS to GD than to G-198. 2/7 patients reported more severe symptoms to G-198 than to GD. 3/7 subjects reported identical severity of symptoms to both apple cultivars.	NA, only 1 dose (15 g)
Bolhaar et al. [28]	5	High allergenic Golden Delicious vs. low allergenic Santana	No information	Mean VAS score after dose 1 (5 g): Santana < GD (*p* > 0.05); Mean VAS score after dose 2 (40 g): Santana < GD (*p* < 0.05); Mean VAS score after dose 3 (120 g): Santana < GD (*p* < 0.05)	“The quantities needed to provoke a similar VAS score were on average 30 times higher for Santana than for GD apples (*p* < 0.001)”
Kootstra et al. [29]	15	High allergenic Golden Delicious vs. low allergenic Santana and Topaz	GD: 1/15 (6.7) Topaz: 1/15 (6.7) Santana: 8/15 (53.5) *p* (Santana vs. GD/Topaz) = 0.002	Maximum VAS score after dose 1 (20 g): Santana < GD (*p* = 0.017), Santana < Topaz (*p* = 0.004)	No information
Vlieg-Boerstra et al. [30]	33	High allergenic Golden Delicious vs. Low-allergenic Elise, Santana and Pink Lady	6–16%; no significant differences between GD, Elise, Santana and Pink Lady (*p* = NA).	Cumulative VAS score after dose 1 (15 g): Elise < Santana (*p* = 0.02), Elise < PL (*p* = 0.04), Elise < GD (*p* < 0.001); Santana < GD (*p* = 0.05)	No information

GD = Golden Delicious; PL = Pink Lady; VAS = visual analogue score; NA = not available.

**Table 6:** GRADE assessment.

Outcome	Study Design	Risk of Bias	Inconsistency	Indirectness	Imprecision	Publication Bias	Overall GRADE
**Oral Immunotherapy**							
Frequency of achieved tolerance to apple at final follow-up [21,22]	3	−1	−1	0	−1	0	VERY LOW
**(Heat) Processing**							
Frequency of (NO) symptoms in DBPCFC with processed food [23,24,25,26]	2	−1	−1	−1	−1	0	VERY LOW
Symptom severity in DBPCFC with raw vs. processed food [23,24,25,26]	2	−1	−1	−1	−1	0	VERY LOW
Dose eliciting symptoms in DBPCFC with raw vs. processed food [23,25,26]	2	−1	−1	−1	−1	0	VERY LOW
**Consumption of Hypoallergenic Cultivars**							
Frequency of NO symptoms to highest dose in FC [27,29,30]	2	−1	−2	0	−1	0	VERY LOW
Severity of symptoms in FC with various cultivars [27,28,29,30]	2	−1	−2	0	−1	0	VERY LOW
Dose eliciting symptoms in FC with various cultivars [28]	2	−1	−2	0	−1	0	VERY LOW

DBPCFC = double-blind placebo-controlled food challenge; FC = food challenge.

## Appendix A

Search strategy for PubMed Search (performed 6 July 2018).

**DOMAIN: Pollen-related food allergy**

1. food*[Title/Abstract] OR fruit[MeSH Terms] OR fruit*[Title/Abstract] OR rosaceae[MeSH Terms] OR rosaceae*[Title/Abstract] OR apple*[Title/Abstract] OR malus*[Title/Abstract] OR apricot*[Title/Abstract] OR cherr*[Title/Abstract] OR peach*[Title/Abstract] OR plum*[Title/Abstract] OR nectarin*[Title/Abstract] OR prunus*[Title/Abstract] OR pear*[Title/Abstract] OR pyrus*[Title/Abstract] OR actinidia[MeSH Terms] OR actinidia*[Title/Abstract] OR kiwi*[Title/Abstract] OR mangifera[MeSH Terms] OR mangifera*[Title/Abstract] OR mango*[Title/Abstract] OR diospyros[MeSH Terms] OR diospyros*[Title/Abstract] OR artocarpus[MeSH Terms] OR artocarpus*[Title/Abstract] OR jackfruit*[Title/Abstract] OR litchi[MeSH Terms] OR lychee*[Title/Abstract] OR litch*[Title/Abstract] OR leechee*[Title/Abstract] OR vitis[MeSH Terms] OR vitis*[Title/Abstract] OR grape*[Title/Abstract] OR ficus[MeSH Terms] OR ficus*[Title/Abstract] OR fig*[Title/Abstract] OR fabaceae[MeSH Terms] OR fabaceae*[Title/Abstract] OR legume*[Title/Abstract] OR soy food[MeSH Terms] OR soybeans[MeSH Terms] OR soy*[Title/Abstract] OR soj*[Title/Abstract] OR bean*[Title/Abstract] OR vegetable[MeSH Terms] OR vegetable*[Title/Abstract] OR daucus carota[MeSH Terms] OR daucus carota*[Title/Abstract] OR carrot*[Title/Abstract] OR apium graveolens[MeSH Terms] OR apium graveolen*[Title/Abstract] OR celer*[Title/Abstract] OR nuts[MeSH Terms] OR nut[Title/Abstract] OR nuts[Title/Abstract] OR corylus[MeSH Terms] OR corylus*[Title/Abstract] OR hazelnut*[Title/Abstract] OR arachis[MeSH Terms] OR arachis*[Title/Abstract] OR peanut*[Title/Abstract] OR solanum tuberosum[MeSH Terms] OR solanum tuberosum*[Title/Abstract] OR spices[MeSH Terms] OR spice*[Title/Abstract] OR herb*[Title/Abstract] OR sunflower seed*[Title/Abstract]
2. hypersensitivities[MeSH Terms] OR hypersensitiv*[Title/Abstract] OR allergens[MeSH Terms] OR allerg*[Title/Abstract] OR cross reactions[MeSH Terms] OR cross react*[Title/Abstract] OR crossreact*[Title/Abstract] OR ige mediat*[Title/Abstract] OR sensitis*[Title/Abstract] OR sensitis*[Title/Abstract]
3. 1 AND 2
4. food hypersensitivities[MeSH Terms]
5. 3 OR 4
6. pollen[MeSH Terms] OR pollen*[Title/Abstract] OR trees[MeSH Terms] OR tree*[Title/Abstract] OR orchard*[Title/Abstract] OR plane*[Title/Abstract] OR betulaceae[MeSH Terms] OR alnus*[Title/Abstract] OR alder*[Title/Abstract] OR betula*[Title/Abstract] OR birch*[Title/Abstract] OR corylus*[Title/Abstract] OR hazel*[Title/Abstract] OR filbert*[Title/Abstract] OR hornbeam*[Title/Abstract] OR quercus[MeSH Terms] OR quercus*[Title/Abstract] OR oak*[Title/Abstract] OR poaceae[MeSH Terms] OR poaceae*[Title/Abstract] OR grass*[Title/Abstract] OR timothy*[Title/Abstract] OR artemisia[MeSH Terms] OR artemisia*[Title/Abstract] OR ambrosia[MeSH Terms] OR ambrosia*[Title/Abstract] OR mugwort*[Title/Abstract] OR ragweed*[Title/Abstract] OR plant weeds[MeSH Terms] OR weed*[Title/Abstract]
7. 5 AND 6
8. oral allergy syndrom*[Title/Abstract]) OR pollen food syndrom*[Title/Abstract]
9. 7 OR 8

**DETERMINANTS: Oral immunotherapy, (heat) processing, hypoallergenic cultivars, dietary avoidance**

10. immunotherapy[MeSH Terms] OR immunotherap*[Title/Abstract]
11. oral[Title/Abstract] AND tolerance[Title/Abstract] AND induc*[Title/Abstract]
12. 10 OR 11
13. heating[MeSH Terms] OR heat*[Title/Abstract] OR cooking[MeSH Terms] OR cook*[Title/Abstract] OR roast*[Title/Abstract] OR baked[Title/Abstract] OR baking[Title/Abstract] OR microwav*[Title/Abstract] OR pasteuriz*[Title/Abstract] OR pasteuris*[Title/Abstract] OR process*[Title/Abstract] OR dehydrat*[Title/Abstract] OR dried[Title/Abstract] OR spice*[Title/Abstract] OR herb*[Title/Abstract]
14. hypoallergen*[Title/Abstract] OR hypo allergen*[Title/Abstract] OR low allergen*[Title/Abstract] OR reduced allergen*[Title/Abstract] OR cultiv*[Title/Abstract] OR variet*[Title/Abstract]
15. diet[Title/Abstract]) OR diets[Title/Abstract] OR dietary[Title/Abstract] OR avoid*[Title/Abstract] or trace[Title/Abstract] OR traces[Title/Abstract]
16. 12 OR 13 OR 14 OR 15
17. 9 AND 16
